# MC Sensor—A Novel Method for Measurement of Muscle Tension

**DOI:** 10.3390/s111009411

**Published:** 2011-09-30

**Authors:** Srđan Đorđević, Sara Stančin, Andrej Meglič, Veljko Milutinović, Sašo Tomažič

**Affiliations:** 1 TMG-BMC Ltd., Splitska 5, 1000 Ljubljana, Slovenia; E-Mails: srdjand@tmg.si (S.Đ.); andrej.meglic@tmg.si (A.M.); 2 University of Ljubljana, Faculty of Electrical Engineering, Tržaška 25, 1000 Ljubljana, Slovenia; E-Mails: sara.stancin@fe.uni-lj.si (S.S.); saso.tomazic@fe.uni-lj.si (S.T.); 3 University of Belgrade, School of Electrical Engineering, Bulevar kralja Aleksandra 73, 11120 Belgrade, Serbia; E-Mail: vm@etf.rs

**Keywords:** biomechanics, skeletal muscles, muscle force, contractile properties, measuring techniques, biophysics

## Abstract

This paper presents a new muscle contraction (MC) sensor. This MC sensor is based on a novel principle whereby muscle tension is measured during muscle contractions. During the measurement, the sensor is fixed on the skin surface above the muscle, while the sensor tip applies pressure and causes an indentation of the skin and intermediate layer directly above the muscle and muscle itself. The force on the sensor tip is then measured. This force is roughly proportional to the tension of the muscle. The measurement is non-invasive and selective. Selectivity of MC measurement refers to the specific muscle or part of the muscle that is being measured and is limited by the size of the sensor tip. The sensor is relatively small and light so that the measurements can be performed while the measured subject performs different activities. Test measurements with this MC sensor on the biceps brachii muscle under isometric conditions (elbow angle 90°) showed a high individual linear correlation between the isometric force and MC signal amplitudes (0.97 ≤ r ≤ 1). The measurements also revealed a strong correlation between the MC and electromyogram (EMG) signals as well as good dynamic behaviour by the MC sensor. We believe that this MC sensor, when fully tested, will be a useful device for muscle mechanic diagnostics and that it will be complementary to existing methods.

## 1. Introduction

Taken together, the skeletal muscles comprise the largest organ in the human body. Furthermore, they consume the most energy of any organ and enable efficient movements of varying intensities and durations in different movement patterns. Tension is one of important skeletal muscle biomechanical properties. The estimation of skeletal muscle tension during contraction is an important element in the daily work in various fields in health and medicine, as well as those that require an understanding of human motion, such as professional sports and physiotherapy.

When performing skeletal muscle tension measurements, it is important to distinguish single-muscle contractions from group-muscle contractions. It is also important to distinguish the activity and establish the tension of a single part of a skeletal muscle, for example, the lateral and medial parts of the gastrocnemius muscle. This process is referred to as muscle measurement selectivity. Furthermore, it is also essential that the measurements are performed in a non-invasive manner *in situ* (*i.e*., the phenomenon should be examined exactly where it occurs).

When establishing skeletal muscle tension, it is also desirable to use the same measuring equipment and to be able to perform similarly efficient measurements when measuring a moving subject, such as during the performance of a sport or activity, or when measuring a still subject, such as when the subject is lying down or sitting while waiting for the measurement to be completed.

The mechanisms that contribute to the decline in muscle force during a voluntary contraction are known to depend on the details of the task that is being performed [1,2]. The task variables that appear to influence the prevailing mechanisms include the type and intensity of exercise, the muscle groups involved and the physical environment in which the task is performed.

Another crucial aspect of the mechanical behaviour of skeletal muscles is the stretch-shorten cycle (the stretch of the active muscle before it performs a shortening contraction) [3,4]. The ability to measure this aforementioned phenomenon in a non-invasive manner in humans has represented a significant challenge in the field, but new approaches can overcome this challenge and contribute to a better understanding of skeletal muscle functioning.

In this paper we present a new muscle contraction (MC) sensor which enables measurements of muscle tension in a non-invasive and selective manner. We evaluated the functionality of the sensor with test measurements on the biceps brachii muscle under isometric conditions (elbow angle 90°). The measurement method and supporting device described in this paper are part of the patent ‘Method and device for non-invasive and selective determination of skeletal muscles biomechanical and contractile properties’ granted by the Slovenian Intellectual Property Office in September 2010 [5].

## 2. Background and Motivation

Current methods for determining different biomechanical and contractile properties of skeletal muscles are wide ranging, and each has its pros and cons. However, these methods predominantly measure only a single component that defines only how a muscle works under the particular test conditions. Furthermore, the majority of tests are conducted in a laboratory [6], are constrained and often involve invasive methods (needle electromyogram, biopsy, maximum force measurement, *etc*.).

The direct determination of biomechanical properties in human skeletal muscles through the estimation of the muscle-fibre-type percentage is usually performed by applying histochemical and immunocytochemical techniques. Both types of techniques are based on myofibrillar adenosinetriphosphatase (M-ATP-ase) activity and myosin heavy chain isoform identification. These techniques are used for samples that have been obtained by muscle biopsy and are therefore considered invasive and not suitable for routine use.

Because of the invasive character of direct methods, muscle function and properties measurements are usually performed through indirect measurement methods that enable the estimation of the strength of a skeletal muscle or a group of muscles.

Biomechanical properties in human skeletal muscles have usually been detected indirectly by measuring muscle force or torque about a specific joint. Clarkson and Gilewich [7] have defined “muscle strength” as a maximum amount of force or tension exerted by a group of muscles or muscle force exerted by a single muscle in a maximum voluntary contraction (MVC) under specific conditions (type of contraction and joint angle). To perform such direct measurements properly, the measuring mechanism would need to be attached to or placed in a muscle tendon. Although such measurements have been performed, they are not suitable for application in clinical or sports settings.

The methods used to measure muscle force or muscle torque still pose technical problems, which have, to some extent, been solved by using devices such as the manual dynamometer, the wire-tensiometer or the isokinetic dynamometer to estimate the mechanical properties of skeletal muscles. Unfortunately, none of these methods offers a dominant advantage that would make it generally applicable.

Measurements performed with the above described devices are non-selective, as it is generally impossible to measure the force or torque of a particular muscle; thus, all in-task active muscles are measured. For example, in a knee extension measurement, the measure force will be a composite generated by the vastus lateralis, vastus medialis, and rectus femoris muscles. Most isokinetic testing machines are sufficient for maintaining a constant angular velocity during testing. However, detailed real-time studies of quadriceps muscles have shown that muscle fibre velocity and the moment arm are basically never constant during this test, which means that it is extremely problematic to interpret isokinetic data in terms of the muscles generating the torque. The results from these types of studies are often vastly overstated and over-interpreted [6]. It is difficult to rigorously interpret torque-velocity data collected in an isokinetic measurement, as a number of factors are typically unknown. These include the following:

1. Muscle physiological cross-sectional area (PCSA) [8];
2. The fraction of the muscle’s PCSA that is activated [9];
3. Absolute moment arm as a function of joint angle and velocity [10];
4. Muscle fibre length as a function of joint angle and velocity [11];
5. Tendon length as a function of joint angle and velocity [10]; and
6. Inertial properties of the joint [12].

These factors are considered independently due to the limitations of isokinetic dynamometers [6]. Different technologies can diminish some of these limitations in musculoskeletal diagnostics. Another large group of devices measures the biomechanical properties of skeletal muscles by detecting body-movement velocity or movements of specific parts of the body. The velocity parameter is present in an individual’s everyday activities and is controlled and regulated during sport activity, and it might change in subjects with neuromuscular system malfunctions. Movement velocity is determined by muscle contraction velocity, which is related to the speed of generating inner muscle tension and depends on muscle fibre composition. Unfortunately, many other factors also impact movement velocity: mass of body segments, muscle length, physical conditions, body and outer temperatures, inner frictions, gravity and other physiological factors. As muscle fibre composition is not the only factor that impacts movement velocity, the relevance of the results obtained can be disputed.

Traditional measurement devices, such as motion capture systems and force plates, are adequate methods of gait analysis, but they have several limitations, such as high cost and lack of portability [6]. Other established methods for the measurement of muscle properties and the impact of musculoskeletal disorders are also limited. Scientists and medical doctors are often interested in muscle functioning. They observe force development during the process of contraction through either indirect (mainly muscle torque) or direct measurements; in most cases, electrical activity is monitored by electromyogram (EMG).

The EMG only provides an interferogram that represents the summated electrical activation pattern of the muscle near the electrode. As muscle force is highly dependent on length (due to the length-tension property) and velocity (due to the force-velocity property), electrical activity alone cannot possibly provide an accurate measurement of muscle force. In addition, as the EMG summates in a way that does not uniquely represent all of the motor units activated, EMG measurements that are used to infer force are highly suspect [6,13–19].

One of the limitations of an interference EMG is the variability in the recording when the same task is performed by different subjects or by the same subject on different days. The two principal reasons for this variability are that the recording conditions change each time the electrodes are attached and that the volume recorded by the electrodes does not cover the whole volume of the muscle involved in the task [20].

The mechanomyography (MMG) measuring method is a relatively new, non-invasive technique that records and quantifies the low-frequency lateral oscillations produced by active skeletal muscle fibres [21–25]. It has been suggested that the lateral oscillations are the result of the following:

- gross lateral movement of the muscle as it moves towards or away from its line of pull during contraction or relaxation;
- smaller subsequent lateral oscillations generated at the resonant frequency of the muscle; and
- dimensional changes of the active muscle fibres [21,23]. The lateral muscle fibre oscillations (quantified at the skin as MMG) reflect the intrinsic mechanical property of motor unit activity.

During dynamic muscle actions, many factors, such as changes in the torque production, muscle length, thickness of the tissue between the muscle and the MMG sensor, motor unit recruitment, and firing rate can influence the amplitude and frequency of the MMG signal [21]. These factors add to a list of mechanisms that may affect the MMG signal, which is considered to be a complex signal even during isometric muscle actions [26]. It is important for future research to continue examining MMG amplitude and frequency responses during both dynamic and isometric muscle actions in an effort to fully assess the potential uses and applications of MMG [21].

The tensiomyographic measuring technique [27] was devised to prevent invasive or indirect measurements of the biomechanical, dynamic and contractile properties of human skeletal muscles. This technique is based on the selective tensiomyographic measurement of muscle belly displacement, in which muscle belly displacement is proportional to muscle force. Apart from its non-invasiveness, selectivity and simple application, tensiomyographic devices also offer high sensitivity, which enables the detection of weak contractions.

## 3. Basic Principle of the MC Sensor

The innovative measuring method and sensor described in this paper enables the determination of skeletal muscle tension through a completely non-invasive *in situ* and selective manner. The method and the device are called the muscle contraction (MC) measuring method and sensor, respectively. The proposed MC method measures the force on the subject’s skin above the skeletal muscle. During skeletal muscle activity, the tension of that muscle changes. Skeletal muscles are able to produce varying levels of contractile force, which induce different levels of tension in the skeletal muscle. The measurement can be performed in a completely non-invasive *in situ* way.

**Figure 1.**
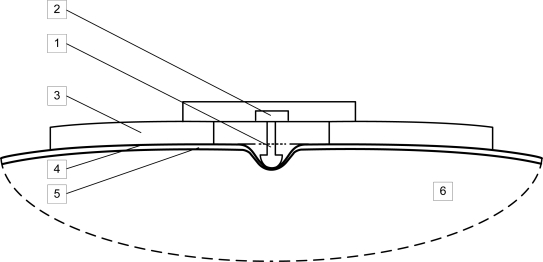
MC sensor for determining the mechanical and physiological properties of skeletal muscles (1): sensor tip; (2): force meter; (3): supporting part; (4): skin surface; (5): intermediate layer; (6): skeletal muscle.

The basic structure of the MC sensor is illustrated in Figure 1. The MC sensor consists of a sensor tip (1), force meter (2), and supporting part (3). The sensor is attached to the subject’s skin surface (4) above the intermediate layer (5) and the skeletal muscle being measured (6).

**Figure 2.**
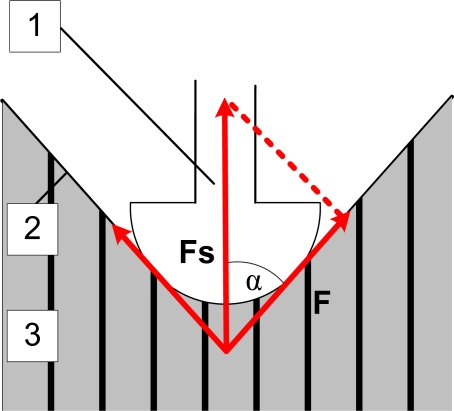
A simplified representation of the MC measuring principle for the determination of the mechanical and physiological properties of skeletal muscles (1): sensor tip; (2): skin and intermediate layer; (3): measured muscle.

The sensor is constructed in such a way that its pressure on the subject’s skin causes the sensor tip to compress the skin surface and the intermediate layer, ultimately placing pressure on the measured skeletal muscle. The sensor tip has to be suitably shaped so it can push down upon the subject’s skin at the appropriate position in a non-invasive way. Any suitable force meter or pressure meter can be used for measuring the force detected on the sensor tip. The supporting part, along with a specially designed attaching part, provides for the suitable attachment and fixation of the sensor on the subject’s skin surface. A simplified (presented only in two dimensions) representation of the principle of the MC measuring method is illustrated in Figure 2.

The measured muscle tension produces the force *F* in the direction along the muscle surface. This force causes the intermediate layer and skin to press on the sensor tip. The vector sum of all forces produces the force *F_s_* in the direction along the sensor tip. This force is then measured with the force meter. In the simplified 2D model presented in Figure 2, this force would be equal to:

$$F_{s}=2F\;\text{cos}\;\alpha$$

where *α* is the angle between the directions of *F* and *F_s_*. In the real world, this equation does not hold exactly. However, as long as the pressure produced by the sensor tip remains constant (*i.e*., the angle *α* between the forces does not change), the measured force is still proportional to the force produced by the muscle tension.

The sensor is attached to the skin of the measured subject through the sensor supporting part. There is also an intermediate layer between the skin and the measured muscle. The thickness as well as the elasticity of the skin and the tissue under the skin differ from subject to subject. Thus, in general, we cannot measure the absolute value of the muscle force without some calibration of the device for each subject separately. However, the dynamical changes of the muscle force can be observed without the need for individual calibration.

When the force on the sensor tip is large, it causes deformation (stretching) of the intermediate layer and consequently diminishes the deepening of the indentation produced by the sensor tip. This reduction increases the angle *α* between the muscle and sensor forces and reduces the sensitivity of the sensor, causing a certain amount of nonlinearity. Finally, if the force is great enough, the deepening would disappear altogether, and the sensor would come to saturation.

The nonlinearity and saturation cannot be determined analytically as they depend on unknown factors, such as the elasticity and thickness of the intermediate layer, which differ from subject to subject. These effects can only be determined empirically by sensor implementation and measurements. The implementation and the measurements are described in the following sections.

## 4. Implementation

**Figure 3.**
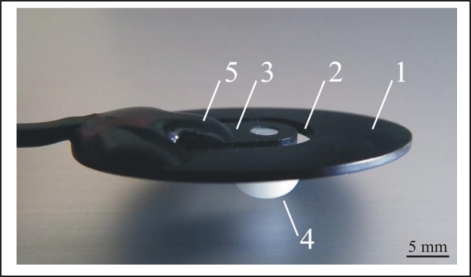
Prototype of the MC sensor (1): laminate; (2): incision; (3): tonguelet; (4): sensor tip; (5): strain gauge.

For test purposes and proof of concept (POC), we implemented the MC sensor as shown in Figure 3.

The elliptically shaped carbon fibre reinforced epoxy polymer (1) was used as the supporting part. A specially shaped incision (2) formed a tonguelet (3), to which the sensor tip (4) was attached (screwed in). Area of the part of the MC sensor, attached to the skin was 650 mm^2^. Area of the sensor tip, applying pressure to the skin was 56 mm^2^. A piezoresistive strain gauge (5) was attached at the root of the tonguelet using a suitable adhesive. As the strain at the root of the tonguelet was proportional to the force acting on the sensor tip, the resistance of the piezoresistor was proportional to that force. To compensate for the temperature sensitivity of the semiconductor strain gauge, the resistance was measured with four piezoresistors connected in a Wheatstone bridge such that all four resistors were placed closely together at the same temperature.

After bonding, the piezoresistor contact pads and cable interface were connected with standard microelectronic golden wire. Silicon piezoresistors were made on phosphorous doped, n-type silicon wafers that were 100 mm in diameter and 380 μm thick with crystallographic orientation and a resistivity of 800 Ω-cm. A special set of photolithographic masks was designed and fabricated. Based on a previous study, the high resistivity boron doped layer was produced through a diffusion process to define the piezoresistor active layer. The diffused pn junction region was covered with a double layer of 20 nm thick silicon oxide and 70 nm thick LPCVD silicon nitride for ambient protection. To ensure electrical isolation between the bonded piezoresistors and retractor, the back portion of the silicon chip was covered with 500 nm thick field oxide and 70 nm thick silicon nitride. The piezoresistors and cable interface were covered with the epoxy casting compound TRA-CAST 3103 to protect the thin interconnection golden wires and to prevent unwanted photo effects caused by exposing the light sensitive piezoresistors.

**Figure 4.**
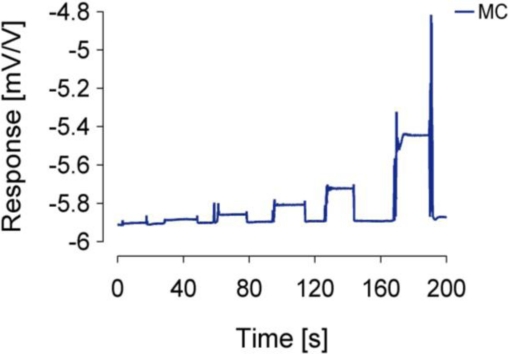
Timeline of MC output response at various weights hanged on the tonguelet.

**Figure 5.**
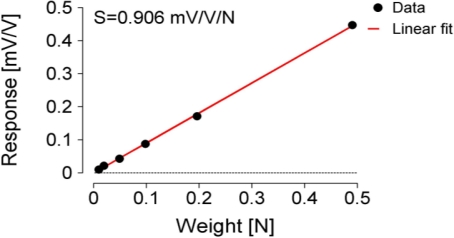
MC sensor sensitivity.

The concrete MC sensor implementation output response (in mV/V), measured at six different weights (1, 2, 5, 10, 20, and 50 g), is presented in Figure 4. The artefacts were caused during manual weights exchanges on the tonguelet. Figure 5 shows that the sensitivity of the MC sensor was S = 0.906 mV/V/N.

## 5. Measurements

The measurements were performed to test the performance of the MC sensor prototype described in the previous section. The measurements were performed mainly to establish the relationship between the force measured by the MC sensor and the force (or effect of the force) produced by the measured muscle. The results were also compared to the simultaneously measured EMG signals for the same muscle.

The EMG and MC signal were measured simultaneously. We assumed that the measurements do not interfere with one another, as the first measurement is electrical and the second is mechanical. On one hand, EMG electrodes were too far from the MC sensor to cause any significant change to the force acting on the MC sensor. On the other hand, the MC sensor was electrically isolated from the skin and could not have any significant effect on the electrical EMG signal. As the muscles were contracted voluntarily, the experiments performed cannot be exactly reproduced. However, separate measurements of EMG and MC signals did not show any qualitative difference from simultaneous measurements.

### 5.1. Methods

#### 5.1.1. Setup

**Figure 6.**
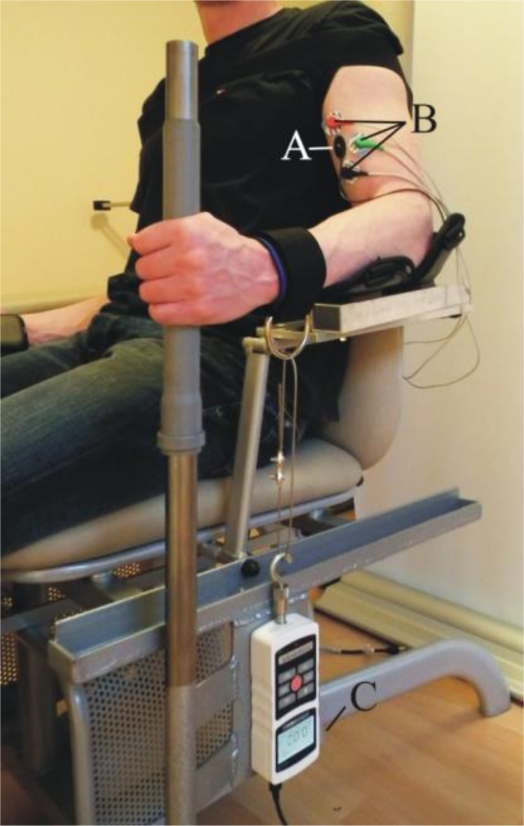
Measurement setup (A): MC sensor; (B): EMG electrodes; (C): force gauge.

The measurements were performed on the biceps brachii (BB) muscle under isometric conditions (elbow angle 90°), as shown in Figure 6.

The subjects were seated facing an LCD monitor with a digital force counter; the non-dominant arm was fixed, and the forearm was in a neutral position. The angle of the elbow joint was approximately 90° and remained constant throughout the experimental session, as shown in Figure 6. The subject’s shoulder on the non-dominant side was securely fastened to the back of the chair to prevent shoulder movement during the voluntary contractions. As the thickness of the tissue under the skin differed from subject to subject, the length of the sensor tip was adjusted for each individual. The adjustment was performed in such a way that at the lowest force, measured with the force meter, we still got enough reading at the output of the sensor and that the subject did not feel uncomfortable. The force with which the MC sensor tip was initially pressed to the subject’s skin was between 0.14 and 0.20 N for all test subjects.

#### 5.1.2. Subjects

Twenty-one males, ranging between 24 and 54 years of age (age 35.1 ± 8.4 years; all values represent the mean ± SD unless otherwise indicated), participated in the study. All of the subjects were healthy and had no known neuromuscular or musculoskeletal disorders at the time of the study. The experimental procedures were approved by The National Medical Ethics Committee of the Republic of Slovenia. All of the subjects gave informed approval before participation in the study.

#### 5.1.3. EMG Recording

A bipolar surface electrode arrangement was placed over the non-dominant biceps brachii muscle [Figure 6(B)]. The interelectrode distance was selected to accommodate the MC sensor. The recording electrodes were placed over the belly of the muscle, approximately midway between the auxiliary fold and the midpoint of the cubital fossa. The reference electrode was placed over the volar arch. The interelectrode impedance was kept below 5,000 Ω through skin abrasion. The EMG signal was recorded using a 24 bit resolution, 25 mV/V NI 9237 module (National Instruments, Austin, TX, USA). We used MLA1010 Disposable ECG electrodes (AD Instruments, Glen Osmond, Australia). To prevent the risk of electric shock, all devices were powered by batteries.

#### 5.1.4. MC Sensor Recording

The MC sensor was placed in the cross point of the longitudinal and transversal meridians of the left biceps brachii muscle [28] [Figure 6(A)]. The signal from the MC sensor was again recorded with the NI 9237 module.

#### 5.1.5. Force Recording

For force measurements, we used the digital force gauge Series 5 (Mark 10, Copiague, NY, USA) with a sampling rate of 10 Hz and a resolution of 0.1 N.

#### 5.1.6. Experimental Protocol

Each experimental session began with the subject performing three trials of MVCs with the elbow flexor muscles. The timing or level of the MVC relied on a verbal count given by an experimenter during which the subject graded the contraction force from zero to maximum in 3 s and briefly maintained this force. The subjects could observe their performance on a monitor with a digital counter and were exhorted to maximise the force during each MVC trial. The subjects were given a rest period of at least 5 min between each MVC. The maximal force exerted by the subject during these three trials was used as the MVC force for the remainder of the experimental session.

Voluntary isometric contractions were performed with the elbow flexor muscles at five target levels: 10, 30, 50, 70, and 90% MVC. For each trial, the target force and the force exerted by the subject were displayed on a monitor with digital force data. The subject was required to match the exerted force with the target and to immediately proceed to next target level until 90% MVC was achieved.

#### 5.1.7. Signal Processing

The EMG signal was sampled at a 5 kHz sampling rate and band-pass filtered at 10–450 Hz. To estimate the amplitude of the EMG, we calculated the RMS value and smoothed the signal using a 10 Hz 6th-order low-pass Butterworth filter. The MC signal was sampled at 5 kHz and then filtered at 10 Hz. The amplitudes of the MC and force (F) were estimated as the differences between the baseline and maximum peak values of the signal.

### 5.2. Results

#### 5.2.1. Example Dynamic Result

**Figure 7.**
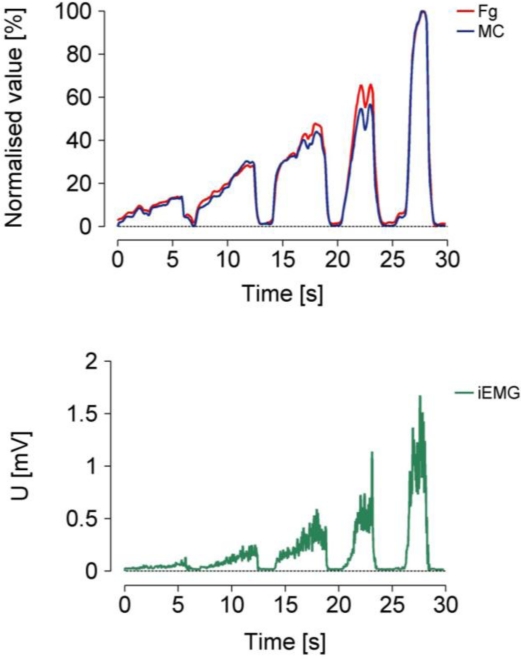
Simultaneous recording of the force (Fg), MC and EMG. The Fg and MC variables are normalised to the maximal value.

An example of the measured MC, integrated electromyogram (iEMG) and force gauge (Fg) signals is presented in Figure 7.

We observed that the normalised MC and Fg (top) values matched closely; this indicates that the response of the MC sensor is nearly linear. The timing of iEMG and MC also matched closely, which indicates that the dynamic behaviour of the MC sensor is comparable to the dynamic behaviour of EMG.

#### 5.2.2. Fg-MC Signal Relationship

The new MC sensor is based on the idea that muscle contractions and tension can be measured selectively and unobtrusively during movement. To determine the relationship between the measured Fg and MC, the signal peaks were compared at 10, 30, 50, 70 and 90% of the maximum isometric contraction (Figure 7). The MC measurements were performed on the BB, which is the strongest elbow flexor, while the Fg measurements were performed simultaneously on the wrist (Figure 6). The elbow angle was fixed at 90°. The strength of the linear relationship between these variables was evaluated with a Pearson product-moment correlation coefficient (r) for each measurement. Very high r values (0.97 ≤ r ≤1) indicate a strong linear relationship between these measured variables. Furthermore, the strength of the linear relationship for all Fg and MC data (21 subjects) was defined by calculating the coefficient of determination (R^2^), which was 0.85.

**Figure 8.**
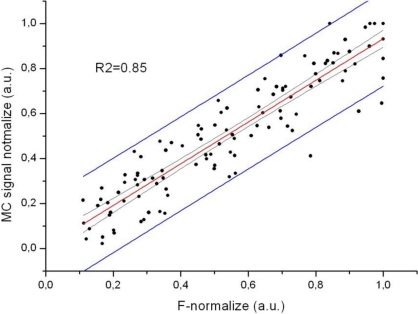
The relationship between force (F) and MC (normalised values). The linear fit is shown with a red line. The grey lines indicate the 95% confidence level, and the blue lines represent the upper and lower 95% prediction limits.

This high individual linear correlation between the force and MC signals was confirmed in each measured subject under isometric conditions. The MC signal was recorded on the biceps brachii muscle. Each calculated r value was between 0.97 and 1. The coefficient of determination, which was calculated to assess the strength of the linear relationship between the force and MC signal for all subjects, was 0.85. Figure 8 shows the normalised Fg-MC signal relationship with a 95% confidence level.

The Pearson product-moment correlation coefficient (r) was also calculated to determine the relationship between the measured MC signal and the iEMG. Very high r values (0.9079 ≤ r ≤ 0.9625) indicated a strong linear relationship between these variables in each measured subject under isometric conditions.

We compared serial isometric muscle contractions (n = 10, 10 repetitions) of biceps brachii and found that the correlation was higher for MC-Fg (0.95 < R < 0.975, average R = 0.96) than for EMG-Fg (0.57 < R < 0.88, average R = 0.77). The standard deviation of the correlation coefficients was also lower for MC-Fg (0.009) compared to EMG-Fg (0.105).

#### 5.2.3. Interclass Correlation Coefficient

Twelve people were measured on two different occasions. Each experiment consisted of voluntary isometric contraction, performed with the elbow flexor muscles. MC sensor was fixed on the biceps brachii muscle. The joint angle was 90°. Values of MC at 10, 20, 50 and 100 N of force, measured with digital force gauge Series 5 (Mark 10, Copiague, NY, USA) with a sampling rate of 1 kHz and a resolution of 0.1 N, were determined. Interclass correlation coefficient for single measures was 0.92 and 0.958 for average measures. Confidence internal was 95%. Statistical analysis was performed using SPSS 90.0 (SPSS Inc., Chicago, IL, USA).

## 6. Discussion

The results of this study indicate that the measured individual F-MC relationship for all tested subjects was highly linear (0.97 ≤ r ≤1) and that MC sensor can be used to determine the relative force of the muscle biceps brachii experimental model under isometric conditions (elbow angle 90°, Table 1).

The strength of the linear relationship between the common force and MC signal data was less significant, but still high. An absolute force relationship cannot be established without precautions and additional measurements. At the very least, the length of the lever arm ‘coefficient’ of the viscoelastic properties of muscle-tendon units and the skin fold elasticity should be incorporated in the final calculations of the absolute force level.

The differences between the individual and total linear relationship among the variables could be attributed to variability in the morphology and length of the forearm, the co-activation of synergistic muscles (e.g., the MC sensor only measures the contraction of the biceps brachii, while the Fg is attached at the wrist; hence, other muscles can also affect the measurement), or to various viscoelastic properties and skin fold elasticity.

The ability to perform non-invasive, selective measurement of the relative force and biomechanical properties of a particular muscle engaged in free voluntary movement engenders the possibility of new applications using complementary methods such as surface EMG.

## 7. Conclusions

We present an innovative method and sensor for selective and non-invasive detection and estimation of skeletal muscles tension. The method was tested for isometric contractions. In the presented protocol, skeletal muscle tension measurements are performed *in situ*. Muscle tension establishment can thus be localised to a specific skeletal muscle part of interest. Selectivity of MC measurement refers to the specific muscle or part of the muscle that is being measured. It is limited by the size of the sensor tip projection on the skin and underlying muscle.

Test measurements were performed on the biceps brachii muscle. High individual linear correlation between the force and MC signals was confirmed in each measured subject under isometric conditions. High linear correlation was also confirmed between the MC signal and IEMG.

The differences between the individual and total linear relationship among the variables could be attributed to variability in the morphology and length of the forearm, the co-activation of synergistic muscles (e.g., the MC sensor only measures the contraction of the biceps brachii, while the Fg is attached at the wrist; hence, other muscles can also affect the measurement), or to various viscoelastic properties and skin fold elasticity. In our future research we plan to test the functionality of the MC sensor on other muscles, in different (non-isometric) conditions, and also during free movement of the test subjects.

## Figures and Tables

**Table 1.** Correlation coefficient r for the force-MC signal relationship. These values were measured simultaneously during isometric elbow flexion (90°) for 21 male subjects on the non-dominant side.

**Subject**	**n1**	**n2**	**n3**	**n4**	**n5**	**n6**	**n7**	**n8**	**n9**	**n10**	**n11**
**r**	0.98	0.99	0.99	0.97	0.97	0.98	0.99	0.99	0.98	0.99	0.99
**Subject**	**n12**	**n13**	**n14**	**n15**	**n16**	**n17**	**n18**	**n19**	**n20**	**n21**	
**r**	0.98	0.98	0.98	1.00	0.97	0.98	0.99	0.99	0.99	0.97	
